# Individualised medicine from the perspectives of patients using complementary therapies: a meta-ethnography approach

**DOI:** 10.1186/1472-6882-13-124

**Published:** 2013-06-03

**Authors:** Brigitte Franzel, Martina Schwiegershausen, Peter Heusser, Bettina Berger

**Affiliations:** 1 Center for Integrative Medicine, Faculty of Health, University of Witten/Herdecke, Gerhard Kienle Weg 4, Herdecke, D-58313, Germany

**Keywords:** CAM, Qualitative studies, Meta-ethnography, Person-centred medicine, Individualised medicine, Personalised medicine

## Abstract

**Background:**

Personalised (or individualised) medicine in the days of genetic research refers to molecular biologic specifications in individuals and not to a response to individual patient needs in the sense of person-centred medicine. Studies suggest that patients often wish for authentically person-centred care and personal physician-patient interactions, and that they therefore choose Complementary and Alternative medicine (CAM) as a possibility to complement standard care and ensure a patient-centred approach. Therefore, to build on the findings documented in these qualitative studies, we investigated the various concepts of individualised medicine inherent in patients’ reasons for using CAM.

**Methods:**

We used the technique of meta-ethnography, following a three-stage approach: (1) A comprehensive systematic literature search of 67 electronic databases and appraisal of eligible qualitative studies related to patients’ reasons for seeking CAM was carried out. Eligibility for inclusion was determined using defined criteria. (2) A meta-ethnographic study was conducted according to Noblit and Hare's method for translating key themes in patients’ reasons for using CAM. (3) A line-of-argument approach was used to synthesize and interpret key concepts associated with patients’ reasoning regarding individualized medicine.

**Results:**

(1) Of a total of 9,578 citations screened, 38 studies were appraised with a quality assessment checklist and a total of 30 publications were included in the study. (2) Reasons for CAM use evolved following a reciprocal translation. (3) The line-of-argument interpretations of patients’ concepts of individualised medicine that emerged based on the findings of our multidisciplinary research team were “personal growth”, “holism”, “alliance”, “integrative care”, “self-activation” and “wellbeing”.

**Conclusions:**

The results of this meta-ethnographic study demonstrate that patients’ notions of individualised medicine differ from the current idea of personalised genetic medicine. Our study shows that the “personal” patients’ needs are not identified with a specific high-risk group or with a unique genetic profile in the sense of genome-based “personalised” or “individualised” medicine. Thus, the concept of individualised medicine should include the humanistic approach of individualisation as expressed in concepts such as “personal growth”, “holistic” or “integrative care”, doctor-patient “alliance”, “self-activation” and “wellbeing” needs. This should also be considered in research projects and the allocation of healthcare resources.

## Background

Rather than referring to “individualised medicine” focusing on individualised care tailored to patient needs, the concept of “personalised medicine” in today’s age of genetic research denotes the molecular biologic specification of individuals [1]. Current statements on personalised or individualised medicine appear mainly in the context of research and academic medicine, politics and economics. In recent years, individualised medicine has become a major research challenge, as clinicians and researchers have sought to discover more specific and individually tailored diagnostic tools and treatments for managing cancer, diabetes, and other common medical conditions [2]. Complementing this increase in genetic and molecular biological knowledge a clear trend has arisen towards genome-based individualised medicine; such genomics-associated discoveries have opened up vast options for health care systems with regard to patient management. The German Bundestag’s recent report on the future of “individualised medicine in the healthcare system” sought to assess the current state of health-related science and technology and the possible developments and implication associated with individualised medicine for medical care, health insurance and companies [2,3]. Therein five concepts of individualisation were presented: (1) individual biomarker-based stratification, (2) genome-based individual health-related characteristics, (3) genetic biomarkers, (4) individual disease risks and (5) differential intervention offerings and unique therapeutic items. As a further delineation, but also as an assignment by definition, “speaking medicine” (i.e. doctor-patient interaction) was attributed to the holistic medical approaches of the Complementary and Alternative medicine (CAM) [3].

However, the question has so far remained unanswered as to whether the current focus of research and academic medicine, politics and economics on molecular biologic specification can ameliorate the healthcare needs of patients in a balanced relation to the invested resources [4,5]. Furthermore, although the aims of innovations in healthcare systems include improved quality of life and other patient-specific goals, healthcare providers often neglect sufficiently to discuss with patients realistic expectations regarding such aims. A research gap has been identified in “that the real target audience for individualised medicine so far has hardly been questioned about their preferences” [3].

Patients’ concerns about this lack of individualised attention and open dialogue have been borne out in a number of reviews suggesting that patients often turn to Complementary and Alternative medicine (CAM) because they feel that the traditional healthcare system does not provide adequate patient-centred care or individualised physician-patient interactions, or because they are seeking more holistic or integrative forms of care [6-8]. The published reviews about reasons for CAM use analyse quantitative studies; at present there is no meta-synthesis of qualitative studies available.

Qualitative studies are applied when methods are needed to understand patients’ subjective experiences and perceptions of healthcare [9-11]. As the nature of clinical knowledge based on quantitative research methods and statistical analysis can be somewhat limited when individual or subjective phenomena, contexts of illness or health, or patients specific individual needs are to be investigated, qualitative methods provide a more thorough approach for describing personal human behaviour and needs; this is also true for the study of CAM [12,13].

Since primary qualitative studies sometimes reveal that concepts of person-centred care are part of the common expectation of patients seeking CAM practitioners [14], it is reasonable to expect that the accumulated knowledge provided by qualitative studies can provide an in-depth understanding as to the concepts, ideas, perceptions, views and expectations of individualised medicine patients have who turn to CAM. For this reason, we decided to explore patients’ views about individualised care by analysing their reasons for seeking CAM and subsequently extract, synthesise and interpret corresponding content from primary qualitative investigations in a meta-ethnographic study.

The goal of the project was to describe the concepts, expectations and perceptions of individualised medicine inherent in patients’ reasons for using CAM, as documented in qualitative studies. To our knowledge, ours is the first publication to address this important research.

## Methods

For this study, the method of meta-ethnography following the style of Noblit and Hare [15] was chosen to collect and analyse the essential knowledge of patients’ reasons for CAM use and to synthesise and interpret patients’ concepts of individualised medicine. A meta-ethnography, a form “pooling” findings of qualitative research, is a meta-analysis with a comparative textual analysis of published qualitative field studies [15]. There remains controversy as to which meta-synthesis method can be best used for diverse sorts of qualitative research projects such as the one described here. In this case, from various methods of meta-synthesis, we determined the meta-ethnography with its interpretive orientation, to be the best approach. Because patients’ concepts, expectations or perceptions of individualised medicine were not readily available in primary studies at the time the research question was raised, we collected and analysed in a meta-ethnography patients’ previously explored reasons for CAM use, and subsequently interpreted patients’ concepts of individualised medicine. The research project included three major sequences (1) a systematic literature search of 67 electronic databases and the subsequent appraisal of selected publications of qualitative studies investigating patients’ reasons for seeking CAM therapies, with inclusion eligibility determined using defined criteria (2) a conduction of a meta-ethnographic study following Noblit and Hare’s [15] method to translate the key concepts of why patients use CAM; and (3) a line-of-argument approach for the synthesis and interpretation of patients’ concepts of individualised medicine.

Following Noblit and Hare, our meta-ethnographic method included seven phases that overlapped and were repeated as the synthesis proceeded (Table 1).

## Results

In the first sequence of the research project a total of 9,578 relevant articles were found, of which 3,615 were screened on the basis of abstracts and titles. Sixty-three full publications were analysed according to the predefined inclusion and exclusion criteria (Table 1). Of these 63 papers, a total of 25 publications were excluded after full text analysis and 38 publications were appraised with a quality assessment checklist. An additional eight publications were excluded following the quality assessment performed by two members of the research team working independently Further details about the literature search results are listed in the Additional file 2. The remaining 30 studies that we synthesised in our meta-ethnography originated mostly in the United States, the United Kingdom or Australia. The majority of these 30 studies consisted of studies of cancer patients or of patients with chronic diseases.

Characteristics of the studies included in the meta-ethnography are presented in Table 2. Of the 30 studies, 27 studies reported results of patients using various CAM modalities. Two of the studies we examined reported the use of meditation and prayer, and one study reported the use of body-based therapies (e.g., massage therapy). Most studies used a qualitative descriptive design and collected data using semi-structured interviews. Study themes were determined to be roughly similar, which Noblit and Hare expressed as “reciprocal” [15].

The reciprocal translation of reasons for CAM use, representing the second sequence of the research project, resulted in the following secondary-order themes: “time”, “holism”, “tailored care”, “teamwork and equal relationship”, “new avenues”, “facilitating transformative effect”, “support for self-healing power”, “gentle and natural treatment”, “less side effects”, “autonomy and active control”, “dimensions of wellbeing” and “accessibility and legitimization”. The translated secondary-order themes were the base for the line-of argument synthesis and the interpretation of patients’ concepts of individualised medicine.

The third sequence of the research project was a “line of argument” synthesis and a higher-order interpretation from the reciprocal translation.

**Figure 1:**
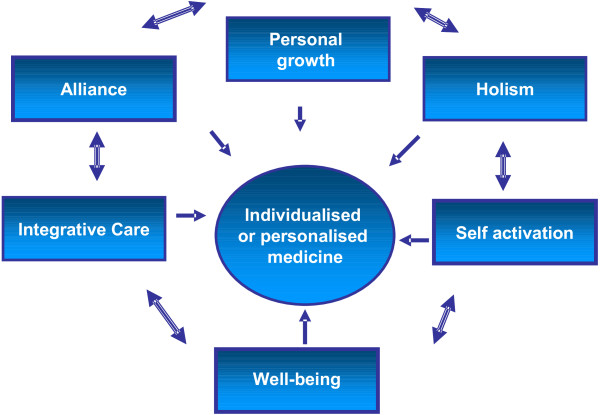
Third-order concepts and their relationship: a model of how patients perceive individualised medicine.

The six third-order concepts interpreted from the data are shown in Figure 1. The synthesis indicates that patients’ value individualised medicine in terms of a humanistic approach, expressing the wish for an opportunity for “personal growth”, a “holistic” form of care, ease of “self-activation” and “integrative care”, a therapist- patient-“alliance” in the sense of establishing a healing relationship and “wellbeing”. These concepts were not exclusive, and they overlapped in certain dimensions and sub-themes. The third-order concepts with the respective dimensions and sub-themes resulting from the “lines-of-argument synthesis” are presented below, with representative quotes from the original papers shown in Table 3.

### Personal growth

Patients’ concepts of “personal growth” stood for a personal transformation process that was expected to be induced or facilitated by the healthcare encounter and that encompassed a reassessment of disease and life histories, an identification of causes, an understanding of the disease, a re-evaluation of attitudes and priorities and a way to find a fitting philosophy of health and life. It also comprised an exploration and implementation of lifestyle changes, including elements such as increased body awareness and spirituality and an appreciation of nature and surroundings. This concept could be further subdivided into the four dimensions described below.

#### Emotional disease handling

Patients’ motivation to seek individualised care and to visit CAM practitioners in the event of a serious or life-threatening illness included the need to find time, space, opportunity and support to interpret and accept the illness emotionally. Here, the emotional and existential consternation caused by disease requires a thorough reassessment of one’s personal situation [16-28].

#### Biographical reassessment

Serious illness often led to questions related to the meaning of life and disease. In their attempt to cope with such questions, people might seek person-centred care to receive assistance. Some patients understood their illness to be a teacher, which could lead to an effort to integrate their disease into the biographical context of their personality [16,17,23,26,29,30].

#### Correlation building

The establishment of a correlation between physical symptoms and psychological, biographical and existential aspects was often understood by patients to be a

**Table 1:** **Meta-ethnography steps according to Noblit and Hare**[15]

		
1	Getting started	“Getting started” meant to define the objective or interest of the synthesis and the wording of the research question [2].
2	Deciding what is relevant to the initial interest	Sixty-seven databases, including medical, social science, psychology, nutrition and complementary medicine databases (i.e., API-on, CAMbase, CAM-QUEST, CINAHL, Cochrane Library, DIMDI, GREENPILOT, Heclinet, MedPilot, PubMed, Psyndex, PsynINFO, Sinbad, Somed), were searched for the Boolean terms “complementary and alternative medicine” OR “CAM” OR “complementary medicine” OR “alternative medicine” OR qualitative research” OR “qualitative studies” OR “interviews” OR “[exploratory OR grounded theory OR content analysis OR focus groups OR ethnography]” OR “reasons” OR “[concepts OR patient expectations OR motivation OR attitude to health OR patient communication OR health knowledge OR patient acceptance of health care OR patient participation OR physician-patient relations OR professional-patient relations]”. The selection of these terms followed predetermined inclusion criteria and included qualitative research articles in English and German about reasons for CAM use from a patient perspective; all articles used in this analysis were published between 1980 and 2011. Exclusion criteria were qualitative studies with therapists, perspectives of teaching personnel, review and theory papers and articles devoted to study design and secondary analysis. A detailed description of the literature search and appraisal of the meta-ethnography will be published separately and is also mentioned in Additional file 1.
3	Reading the studies	The studies were reviewed multiple times, while the findings of the individual qualitative studies were collected with extensive attention paid to the details in the articles and the key themes from each article were determined. Two members of the research team extracted the themes of the individual qualitative studies concerning patients’ reasons for CAM usage and transferred them into a spreadsheet program as primary themes with their related explanations. The spreadsheet’s columns contained the original authors and the key primary themes of reasons of patients seeking CAM, and the rows displayed the main explanations of the key themes or citations of the patients. Key themes were juxtaposed, with the most important interpretations of the authors focusing on concepts of individualised medicine (mostly in the discussion section of each article) in the last column; our team worked diligently to always keep in mind the research question, which was the expectation of patients related to individualised medicine. After the extraction of key themes with reasons of patients for CAM, the spreadsheet data and personal notes were discussed in regular meetings. This discussion revealed no important differences in the extracted data. The consolidated spreadsheet data were finally discussed with the entire research team.
4	Determining how the studies are related	For the syntheses, we had to determine how the individual studies were related. According to Noblit and Hare, the metaphors, concepts or constructs used for this purpose can be either (1) directly comparable as “reciprocal” translations; (2) stand in relative opposition to each other and are essentially “refutational”; or (3) present a “line-of-argument” rather than a reciprocal or refutational translation [15]. Here, “reciprocal” means that the studies can be combined such that one study can be presented in terms of another. “Reciprocal translation” involves uniting ideas and concepts from the original studies through a process of comparing across the studies. “Refutational” means that the studies can be set against one another such that the grounds for one study’s refutation of another become visible. A “line-of-argument” synthesis ties the studies to one another and informs how the individual studies go beyond one another. At the end of this phase, the team assumed that the studies had re-occurring themes and that a “line-of-argument” analysis could be performed.
5	Translating the studies	Translation in a meta-ethnography such as ours means comparing the metaphors and concepts in one article with the metaphors in others. We first arranged all papers chronologically and according to main indications. Thereafter, we compared the key themes from paper one with paper two, and the syntheses of these two papers with paper three, and so on. The translation respected the individual meaning and maintained the central metaphors in relation to the studies’ other key metaphors. We translated our key themes across all articles in order to determine secondary key themes. All secondary key themes contributed reasoning behind why patients turn toward CAM. To perform the translation, the research team members worked with grids or hand cards. The relationship between the studies was indicated by drawing arrows, lines and bubbles or by clustering the hand cards. The emerging secondary key themes were transferred into the head line of a spreadsheet named “secondary key themes,” and the applicable explanations were entered in the rows below, the themes were juxtaposed with the authors’ main secondary interpretations from the discussion section of each article. We made analytical and reflexive notes during the translation to be prepared for the research group discussions.
6	Synthesizing translations	The secondary key themes of the reciprocal translation were brought together by synthesizing them, starting from the identified secondary key themes and matching them with their respective patients’ quotations of the primary studies. This process involved further re-readings of the original studies. The findings from the translation and the resulting spreadsheet data with secondary themes, explanations, interpretations and subthemes provided the foundation for a third order analysis. In this phase it was possible to re-conceptualize the findings, generating a new interpretation of the secondary-order themes. Each member of the research team independently developed an overarching mind-map and his or her own synthesis model that linked together the translated secondary key themes and authors’ interpretations. These models were merged and discussed. In this phase we also used hand cards to pick apart the original explanations of the authors and subsequently put them together again in clusters. The clusters were compared to each other and classified, resulting in our new third-order concepts with dimensions and subthemes. This process was quite similar to standard primary qualitative research in terms of subjectivity of interpretation, and can be compared to a grounded theory approach that puts the similarities between studies into an interpretive order according to Noblit and Hare a “line of argument”. The line of argument synthesis involved building up a picture of the whole from studies of its parts. Our interpretation aimed to develop a model to explain the overall concepts of patients about individualised medicine.
7	Expressing the synthesis	According to Noblit and Hare, the synthesis is mostly expressed in written words or in another presentable form [15]. We created a diagrammatic model and use this for publication and poster presentations to express the synthesis. Quotations were used for validation.

 refreshing exercise and could be perceived as person-centred care when the therapist provides the time and support for such discussion during the patient’s visit [16,17,19-24,26,29].

#### Transformation

The dimension of “transformation” reflected the possibility of personal development and a transformation of life; here, spiritual aspects seem to have become more relevant to patients reporting this dimension [18,27,30,31]. Patients appreciated an individualised approach in which they experienced support in inner development and which could have redefined their position from recipient (of treatment) to that of an explorer as their disease progressed [16,17,19-28]. With person-centred care patients felt empowered to develop new directions for improving their lives and lifestyle [17,26,28,30].

### Holism

The most common theme among all 30 studies was that of “holism”. An individual approach was identified by CAM patients with a whole-person approach or a holistic approach. Instead of singular accounts for biomedical factors and isolated symptoms, patients reported that healthcare providers should take into consideration a wider range of factors or causes based on patients’ opinions; these concerns included a variety of physical, psychological, spiritual, social and economic factors. Most patients acknowledged a wide concept of care, which opens up a greater number of dimensions than pure pharmacological treatment alone. This concept could be further subdivided into the two dimensions described below.

#### Interdependencies of various treatments

Holism reflects a comprehensive account of various levels of treatment. An individualised therapeutic approach could include various interactions with different medical specialties (e.g., surgeon, radiologist, general practitioner, psychologist, physiotherapist) and patient lifestyle aspects such as nutrition and exercise therapy [16-18,21,24,29,32-37].

#### Respect of the whole person’s state

Patients acknowledged the importance of respect of their whole person’s state, specifically referring to their desire for “an individual approach to be seen as a whole person” [19] rather than as composites of various biomedical attributes or isolated symptoms. Likewise, patients expected their therapists or physicians to approach them with a broad holistic world-view that integrated their physical, psychological, spiritual, social and economic dimensions of life.

### Integrative care

Here, the concept of individualised medicine merges with integrative care. “Integrative care” refers to the patients’ need for choosing amongst different treatments options, including treatment alternatives offered by conventional medicine (COM) or combinations with CAM modalities. Patients had the desire for unique treatments that suited them personally, specifically through the option of selecting from a wide variety of modalities. Patients also wished to be explorers of their own health, capable of deciding for themselves among various CAM and COM modalities.

In the majority of cases, patients sought conventional treatment of their disease and appreciated the advances of modern medicine. However, they also wanted to have room for integrating into their care different models or healthcare options. This type of personal problem-solving or coping strategy using both complementary and conventional methods highlighted patients’ willingness to seek out individualised opportunities. Over and above that, integrative care reflected patients’ desire for better access to CAM therapies. This concept also represented patients’ desire to discuss CAM use openly with COM providers without being dismissed or not taken seriously. The “integrative care” concept could be further subdivided into the dimensions described below.

#### Tailored care

Patients wanted their individual life and disease situation respected with a person-centred treatment approach which suited their specific personal needs in diagnosis, risk information and treatment. They appreciated providers’ attempts to match appropriate practices and treatments to their unique problems, values, preferences and life circumstances, including conventional and complementary methods [18,26,28,30,32,33].

#### Integration of CAM and COM

Patients perceived the establishment of a treatment protocol involving CAM as a highly individualised process [16,26,34-36]. However, patients also felt a responsibility to investigate for themselves potential side effects of recommended medications and treatments and through CAM they sought out treatment options that

**Table 2:** Main criteria of included studies

**Author:**	**Indication:**	**Data collection:**	**Objective of each study:**	**Setting:**
**Barrett et al. March 2000 [**[37]**]**	Primary Care	17 patients, semi structured in-depth interviews	To investigate knowledge, attitudes …of patients of CAM.	Madison telephone listings, USA
**Richardson et al. June 2004 [**[19]**]**	Primary Care	204 patients, qualitative comments in health questionnaire	To assess expectations of patients who use CAM	British NHS outpatient department
**McCaffrey et al. July 2007 [**[29]**]**	Primary Care	37 patients, focus group	To identify the motivations of people who choose IM	Integrative care clinic in Cambridge, MA
**Smith et al. May 2009 [**[32]**]**	Primary Care	19 patients, telephone focus group	To explore the attributes of the therapy encounter	New Zealand, clients of massage therapist or practice
**Grace et al. Sept. 2010 [**[20]**]**	Primary Care	22 patients, hermeneutic phenomenology: case studies, focus groups, key informant interviews	To understand the contribution integrative medicine can make to the quality of care	3 integrative medicine clinics in Sydney, Australia
**Nichol et al. Feb. 2011 [**[18]**]**	Primary Care	12 patients, focus groups	To examine the family as a context for beliefs, decision-making about CAM	Family Focus Clinics from Avon Longitudinal Study of Parents and Children sub-study, UK
**Shaw et al. June 2006 [**[21]**]**	Asthma	50 patients, semi-structured interviews with 22 adults and 28 children	To investigate why and how patients and parents of children use CAM	2 contrasting general practices, one in an affluent suburb one in a deprived inner city area, Bristol, UK
**la Cour et al. Dec 2008 [**[22]**]**	Rheumatic Disease	15 patients, in-depth interviews	To investigate patients’ experience and perceptions of CAM	patient-driven rheumatic disease societies, Denmark
**Richmond et al. May 2010 [**[45]**]**	Hepatitis C	28 patients, semi-structured interviews	To describe reasons for the use of mind-body medicine	liver clinic, tertiary healthcare facility in the United States
**Salamonsen et al. July 2010 [**[23]**]**	MS	2 patients, of 12 qualitative interviews, issue (theme)-focused analysis on two cases	To obtain knowledge and understanding on MS patients' experiences related to their CAM use	selection based on Registry of -exceptional Courses of Disease, Norway and Denmark
**Boon et al. Sept. 1999 [**[35]**]**	Breast Cancer	36 patients, focus groups	To explore breast cancer survivors’ perceptions and experiences of CAM	tertiary care allopathic medical centers, Canada
**Canales et al. Jan. 2003 [**[30]**]**	Breast Cancer	66 patients, focus groups	Specific reasons breast cancer surviviors reported for using CAM	Vermont Mammography Registry, Vermont Canada
**Adler, Sept. 2009 [**[25]**]**	Breast Cancer	44 patients, semi structured interviews	To address older breast cancer patients’ seeking of concurrent care	1593 breast cancer case listings provided by the Northern California Cancer Center
**Mulkins et al. March 2004 [**[27]**]**	Breast, Colon, Prostate, Lung and Throat Cancer	11 patients, unstructured interviews	To identify features of the transformative experience among people who are seeking integrative care	3 integrative care facilities in Vancouver
**Steinsbekk et al. Febr. 2005 [**[38]**]**	Breast, Kidney, NHL, Melanoma, Colon….	17 patients, semi structured interviews	How patients experience consultations with CAM practitioners	outpatient clinic of oncology department at the university hospital, Norway
**Singh et al. Febr. 2005 [**[41]**]**	Prostate Cancer	27 patients, semi structured interviews	To compare the perceptions, beliefs, ideas and experiences that contribute to use CAM	part of a larger study, Hawaii Tumor Registry, USA
**Ribero et al. July 2006 [**[26]**]**	Breast CA	6 patients, semi structured interviews	To describe the attitudes, beliefs and utilization of CAM	Komen Hawaii’s Race for a Cure
**Correa-Velez et al. Oct. 2005 [**[43]**]**	Advanced cancer	39 patients, semi structured interviews	To identify in detail the reasons for using CAM	records of state cancer registry, Queensland, Australia
**White et al. June 2006 [**[16]**]**	Prostate cancer	29 patients in-depth interviews?+?focus groups, then secondary analysis from 10 of 29 patients with spiritual practices	To assess decision making by men who use CAM	men with a confirmed diagnosis of prostate cancer in British Columbia and Alberta, Canada
**Humpel et al. Sep. 2006 [**[39]**]**	Breast. Prostate, colon, lung, liver cancer	19 patients, semi-structured in-depth interviews	To gain a greater understanding of CAM including motivations	recruited via posters and study flyers placed in med. waiting rooms, Australia
**Evans et al. Jan. 2007 [**[17]**]**	Prostate, lung colorectal…	34 patients, semi-structured interviews	To investigate why men with cancer choose to use CAM	National Health Service (NHS]oncology unit, NHS homeopathic outpatient, private cancer charity
**Jones et al. March 2007 [**[36]**]**	Prostate Cancer	14 patients, semi-structured interviews	To examine the cultural beliefs and attitudes of the use of CAM	Prostate cancer center in central Virginia?+?referred by other participants, USA
**Broom August 2009 [**[28]**]**	multiple indication cancer	20 patients, semi-structured interviews	To question how individuals make sense of diverse treatment practices	two oncology departments in Australia
**Wanchai et al. July 2010 [**[31]**]**	Breast Cancer	9 patients, in-depth interviews	What were the breast cancer survivors’ perceptions about CAM	Cancer Center in the Midwestern region of USA
**Foote-Ardah July 2003 [**[44]**]**	HIV	62 patients, qualitative interview, mostpart conversational	To aid understanding why people us CAM for HIV	Core group of persons withHIV from personal networks and contacts made through fieldwork, USA
**Chen et al. May 2009 [**[46]**]**	HIV	29 patients, semi-structured, in-depth interview	To explore issues related to attitudes toward CAM	Ditan hospital in Beijing, China
**McDonald et al. Oct. 2010 [**[40]**]**	HIV	9 patients, semi-structured interviews	To examine the sociocultural meaning and use of CAM	Referrals from CAM practitioners at community-based health service for PLWHA, Melbourne, Australia
**Walter et al. May 2004 [**[33]**]**	Menopause	36 patients, focus groups, and 4 semi-structured interviews	To examine patients’ perspectives of risk communication	two Cambridge practices from contrasting parts of the city
**Patterson et al. Jan. 2008 [**[34]**]**	Primary Care	13 patients, semi-structured interviews, adolescents 15–20 years	To explore adolescent CAM use	Canadian College of Naturopathic Medicine
**Conboy et al. 2008 [**[24]**]**	Endometriosis	7 patients, semi-structured interviews, adolescents 13–22 years	To understand experiences of adolescents with acupuncture	primarilythrough the Division of Gynecology of Children’s Hospital, Boston, MA

 included fewer side effects, even when this interest was not shared by COM practitioners [29].

### Alliance

One commonly identified expectation concerning individualised care expressed by patients who sought help from CAM therapists was the wish for a caring doctor-patient “alliance”. This concept could be further subdivided into the two dimensions described below.

#### Time

A number of papers mentioned time as an important concept related to patients’ perceptions of individualised care of CAM therapists. Specifically, patients wanted the undivided attention of their physicians, individual one-by-one time, the possibility to get additional appointments also in between regular visits, time to think about different treatment options seriously before making a decision and the feeling of the individual of being listened to [19,32,33,37]. Patients reported that they wished to have sufficient time for telling their personal history and for discussing health issues and for asking questions and obtaining appropriate explanations about disease and treatment options [17,19,20,22,27,32,33,37],[38].

#### Healing relationship

Patients expected respect from their physician [18,25,33,37-39]; they also asked for guidance [19,24,32,34,36,40] and expressed a desire for an emotional bond with their care providers. The establishment of an effective doctor-patient “alliance” was directed towards a common goal; avoided paternalism and stereotypes; included an engaged and caring, empathetic and non-judgemental attitude on the side of the practitioner; and allowed for deeper patient understanding and empowerment [17-19,21,24-27,30,32,34-36,39,40].

### Self-activation

Patients’ perspective on individualised medicine and their desire for “self–activation” represented the empowerment of “personal autonomy” and the “activation of the self-healing power” of the patient. Here, “personal autonomy” referred to the patient’s conception of himself or herself as a victim (i.e., an ill person) as opposed to that of a person who has (re-)gained control over their treatment and health.

#### Personal autonomy

The dimension of gaining or regaining “personal autonomy” described patients’ wish to be enabled through an individualised approach of “educational empowerment” to cope with and accept their own health and medical condition; take responsibility through active control for their own health; and become actively involved in decision making related to their condition [17,22,23,27,38,41].

#### Activation of self-healing power

Several patients were persuaded that the activation of self-healing resources might have physiological, psychological, social, spiritual and quality-of-life benefits. Patients wanted to support their individual self-healing capacities and subsequently apply CAM to the standard treatment they received. This corresponds to the salutogenic idea [42] referring to approaches that support healing processes and wellbeing rather than fighting factors that cause disease. Correspondingly, patients wished for their health care providers to support, guide and coach them in developing and using self-healing techniques. Patients believed that individualised healthcare enforcing psychological processes which facilitated hope, positive expectations and feelings, relief of anxiety and anticipation of improvement could influence physiological processes and contribute to healing over and above pharmacologically mediated processes [17,22,23,27,30,35,38,39],[43].

### Wellbeing

Patients expressed a basic need for health and appreciated the benefits of COM options for ameliorating and curing disease. However, patients at times experienced disappointment with COM and subsequently sought out alternative treatments. In the 30 studies synthesised here, patients turned to CAM and used alternatives mainly as individually tailored complements to standard medical treatments in a sense of “wellbeing” or quality of life. Here, “wellbeing” as a concept of individualised medicine reflected patients’ wish for a physically and psychologically healthier feeling, emotional clarification and the relief from chronic symptoms. This concept could be further subdivided into the four dimensions described below.

**Table 3:** Patients’ concepts of individualised medicine

**Concepts**	**Dimensions**	**Sub-themes**	**Quotations**
Personal growth	Emotional disease handling		“I know that a cancer diagnosis is very dramatic. It changes your life forever. It makes you realize that you are mortal. It is only those people who have serious illnesses early in their life who are forced to stop and look at the fact that their life is so fragile. Nobody knows how much time you have left. Somewhere along the line I decided that I was going to use this as an opportunity to strengthen myself. I guess to take charge and get rid of all of this baggage I have been carrying around for the past 20 odd years or so” [27]. “I would say sometimes that a trauma like cancer is a blessing in disguise because it makes you realize to live each moment. Each moment is precious” [16].
	Biographical reassessment		“I don’t sweat the small stuff anymore. Life is too short” [26]. “Maybe it [breast cancer] was just a blessing in disguise” [26]. “I mean, I changed my whole thinking. I was all into my career, and then I thought, do I want the kids to remember me as going out the door all the time or making chocolate chip cookies. And I totally reversed my thinking and stayed home for quite a few years, 3 or 4 years. I was more of a housewife and mother and all that. I don’t regret that because I have three lovely children” [30].
	Correlation building		“For the first time I felt like the various and seemingly disparate symptoms I was coming in with actually made sense to my healthcare provider and fit within a framework that that person understood, and also within a treatment model that that person understood, and then could be used to help make me better—which it is, and I am” [29]. “Today I see that heavy mental pressure over time was what set the MS off, so preventing stress is my best medicine” [23].
	Transformation		“kind of like an abstract thing if you feel within yourself. How do you put that? I think I developed a stronger love for nature and the world around me – that kind of religious. Not, ‘oh – God saved me.’ I got more in tuned with my environment” [26]. [She judged the effectiveness of her therapy both in terms of symptom relief and support for a] “wider transformative journey” [26].
Holism	Interdependencies of various treatments		"It's time in the sense that they have got longer, but also they appear to be more interested. I like our GPs enormously and they're very talented individuals, but they don't have the time to talk…(homeopaths) look at the whole thing and they will say about diet, they will say what about your bedding, what about this, have you changed that?" [21]
	Respect of the whole person’s state	Physical/Psychological holism	“When I am feeling good, I think it’s mental and it’s physical and it’s spiritual; it’s all of it together” [29].
			“I think it’s healing emotionally, and when it healed you emotionally, it healed you physically” [31].
		Spiritual holism	“And so preventative medicine, good, and I mean,…I think a very holistic view is good, and so if something helps you, even though it might seem rather mystic or mystical and you know, I think try it, although I am from a medical background and…on one hand I’m thinking well, we need to see research,…and I work in a very research-based kind of environment, but I’m also a great believer in…these other kinds of metaphysical or other kinds of therapy in any situation” [18].
		Social holism	“…I go already relaxed knowing that it is going to be a really useful hour, that she is interested not just in what I might be feeling or the things I think could need working on but interested in what has been going on in my life [She] knows a bit about my family, my background she knows where there might be problems areas outside of the body and this will help to create al feeling of trust and you can rely on it and rely on her. She does the things like the glass of water and the personalised stuff and oils and what have you. It’s just knowing that you will go away feeling that you’ve had both physical and emotional support” [32].
		Economical holism	“It’s not cheap, but I find I get benefit from it. So, I spend my money for something like this” [31].
Alliance	Time	Time to be listened to	“I think the biggest thing is that there is time. There is individual, one-on-one time” [29].
			“I think the quality of listening is very important. My experience [with IM] has been that the doctors listen, and they make suggestions, and they listen back to how you feel about the suggestions. I am beginning to think that progressive medicine is finding a doctor who will listen” [29].
		Time for transformation	“You know in retrospect, it all looks so obvious. Now I see so many people who I feel are stagnant. It is a matter of being ready to embrace all of this chaos. This kind of self-involvement won’t happen unless you are 100% into it. It has been my own personal journey, and looking back, I don’t think it would have happened any sooner. You truly need to be ready to take it on. Once you are I guess maybe things just start to happen” [27].
		Time between and during visits	“The doctor sees you for certain periods of time and they leave you alone in between” [26].
	Healing Relationship	Respectfulness	“And every time I bring it up they blow it off. So I didn’t get very far when I voiced my concerns.” [37] “Yes, perhaps there’s a difficulty now between being autocratic and being patronising, which must be quite though” [33].
		Wish for guidance, counselling and empowerment	“…it’s a partnership, they’ll look at what can you do as well” [32]. “To be advised and encouraged and to be made aware of how I can improve and help myself. To reach a better state of health and also mind” [19].
		Emotional bonds	“My doctor here, she was funny, graceful, and loving and so she empowered me. We make decisions here as equals. She said, “Okay, so what do you want to do?” It was like I was the doctor. And so I told her some things and she said, “Yeah, okay, I agree with that.” She was just so clear. She was always there for me too. In all of my care experiences here, it was like, “Tell me what is going on for you. Okay, well here are a few things you might want to try and this is what you can expect” [27].
Integrative Care	Tailored Care		“Because people are individuals, it could suit some people a lot better than like, mainstream medicine, and…some people may just be more comfortable with that.…I think homeopathic medicines are a good…rather than just like the same thing for each different illness sort of thing, something unique for each person that suits them. I think…that works very well, so yeah” [18].
			“I would consider one [risk information] that’s more tailored to the individual, instead of being given books that say ‘The risk is this, the risk is that.’ It’s too general. Why isn’t it tailored for the person who’s there? Instead it’s blunderbuss approach really, it’s just kind of so wide” [33].
	Integration of CAM and COM		“Making a decision about what treatment to go for is a combination of belief, what you feel in your own body, and whether others have had success. That’s what drives me . . . if you rely on one doctor, or whoever, you only get part of the picture. In the end only you can bring all the elements necessary together to make a decision.” [28] “I like seeing a doctor who is aware of the bigger picture. Even if she decides or recommends a conventional treatment, at least I know they’re aware of alternative health thinking…that gives me more confidence in the treatment, even if their treatment might end up being the same [conventional]” [29].
	Accessibility		“I expect it [CAM] to be provided on the NHS and [to be] more widely available” [19].
			“I don’t think they [oncologists] were terribly encouraging. I suppose . . . I know complementary medicines work, but I had this horrible thing with my diet I was doing with nuts and fruit. When I told him what I was doing all my doctor said to me was, ‘What do monkeys eat?” [28].
	Legitimating alternatives		“It is just as sound as conventional medicine. It’s just that there haven’t been enough studies yet” [37]. “CAM needs to be looked at scientifically in order to give it the credibility that it deserves” [26].
Self Activation	Personal autonomy	Empowerment through education and counselling	“I went to seminars where there was a group of people that offered different thoughts about food as alternative medicine. It was very interesting and very much an education. I also read a lot and talked a lot to herbalists and naturopaths” [31]. “In the past, in [conventional] medicine, only the doctors go to lectures, to learn new cutting edge things. But we’re all trying to find out what’s on the cutting edge now. We’re our own physicians” [29].
		Active control	“I know that my disease course is unusual. If I had given away the responsibility and taken cortisone and let myself be controlled, I would have been in a totally different place.//If I hadn’t taken all these alternative therapies and walked the road I have walked, I would have been in a wheelchair a long time ago.//In the end we are our own teachers and masters . . . I feel that I’m starting to own more of my story even though a lot is still too painful to relate to” [23]. “At least I felt I was in control and trying to do something to help myself, which made me feel better” [18].
	Activation of self healing power	Activation of physiological self healing	“I think that the two basic differences in approach are: 1) attack the disease, the problem itself, or 2) support the body to attack it. And those are the two different approaches. I compare the medical approach at the moment to the napalm bombing of Viet Nam. I think that is the kind of mind set—we have a problem and we’re going to eradicate it. . . . What are you aiming at: Do you want to kill the cancer cell or do you want to strengthen my body?” [35]
		Healing power of mind	“I think your overall spiritual, psychological state has a lot to do with [disease] progression. If you believe the thing is more powerful than you are or somehow able to inflict great damage, it’s like pointing the bone. But if you can…get the thing into perspective and say it’s just a chronic thing then I think it doesn’t progress as fast” [40]. “I think so yeah because your mind can influence your body so I think that if you don’t believe that it’s going to work then it won’t work” [34].
Wellbeing	Physical wellbeing		“As I have had ankylosing spondylitis for over 30 years and angina for about 7 years, I do not expect to be cured. But I hope that my back and pain from my frozen shoulder which I had for 4 month since my retirement at age 65 will be reduced enough to enable me to enjoy my gardening and an occasional round of golf” [19].
	Psychological wellbeing		“…feeling comfortable whether it’s the physical state of the room or the, the welcome of the therapist, all, all does something to lower those barriers and make you feel more open and trusting” [32].
	Avoidance of adverse drug or treatment effects		“I’m sure you heard one time, ‘The treatment is worse than disease,’ you know, before it becomes an advanced disease. All the side effects that you experienced from the Western medicine treatments. Oh, my God, can there be a better way to treat?” [31]
	Wellbeing after emotional clearing		“For the first time I felt like the various and seemingly disparate symptoms I was coming in with actually made sense to my healthcare provider and fit within a framework that that person understood, and also within a treatment model that that person understood, and then could be used to help make me better—which it is, and I am” [29]. “It was sometimes really hard to get in there and break it all down to look at what I am made up of, at a microscopic level. It gave me this new appreciation as to why I am the way I am and why I react the way I do. Before I knew if I felt sad or scared, but never really totally explored why. Like really explored. It is a tough thing to do” [27].

#### Physical wellbeing

The dimension of maintaining physical wellbeing and functionality (i.e., being more active and continuing to work during treatment) was of great importance to patients. This dimension was related to the life limitations which an illness can cause and to patients’ hope for possible improvements brought about with the support of individualised medicine [17,19,20,26,34,43].

#### Psychological wellbeing

Patients sought a treatment environment in which they were able to relieve their tensions. CAM therapists were perceived as making a greater attempt than COM providers to individualise care so that patients could experience a relaxed, supportive environment that also attended to the purely hedonistic aspects of patient care (e.g., relaxing environment with music); also important to this experience was the provision of therapeutic CAM-based care throughout the patient encounter. Patients construed this form of individualised care as not causing stress and as being enjoyable, and as providing the opportunity for a “time out” from regular activities [17,19,24,26,27,32,39,43-45].

#### Avoidance of adverse drug or treatment effects

This dimension of “wellbeing” denoted the wish of patients for individualised natural treatment with fewer side effects. Furthermore, patients wanted an individualised approach that included CAM treatments as a natural strategy to deal with harmful treatments and to relieve the side effects, damage and discomfort caused by conventional treatments [17,19,22,23,26,30,34,35],[39,41,43,44,46].

#### Wellbeing after emotional clearing

Some individualised healthcare modalities that triggered self-regulation were perceived by patients as being stressful at the outset, but patients expressed that they subsequently experienced a pleasing effect once they had successfully navigated this temporarily emotional exertive situation. “Wellbeing” was not synonymous with pure wellness in a hedonistic sense only, but resulted from deeper conflict solving, in a sense of personalised care [17,18,27,29].

## Discussion

This meta-ethnographic study used the three-stage approach of a rigorous literature search and quality appraisal, a synthesis of qualitative research and an interpretation of overarching constructs for addressing the research question as to what concepts of individualised medicine patients have who use complementary therapies. Although there exist a handful of research projects with qualitative studies that begin to investigate patients’ notions of personalised medicine, [47-49] the relative dearth of primary studies reporting on this topic required us to take the indirection with reasons for CAM use as documented in qualitative studies.

With a meta-ethnographic methodology, our synthesis could proceed from a reciprocal translation of reasons for CAM use to a higher-order interpretation in the same way that a primary study might move from a descriptive analysis to an explanatory analysis [50-53]. Meta-ethnography such as in our study can also be used for understanding and enriching the discourse on humanistic issues [15,54-56].

Other published meta-ethnographic studies differ in their methodology with regard to the steps we described above. In this project, we tried to stay as close as possible to the methods suggested by Noblit and Hare [15]; however, our procedure may add ideas and material for further clarifications in the development of the meta-ethnographic synthesis procedure.

As is common in qualitative research projects, a key question appeared as to when and how data saturation was achieved. We discovered that after translating two-thirds of the studies, no new themes could be found; we even went so far as to extend this synthesis to the excluded studies to provide the most robust analysis possible, with the same result. Previously published reviews of specific and individual-preferences in healthcare and patients’ reasons for turning to CAM report results that are somewhat comparable to those of our second-order constructs of our meta-ethnographic study [6-8]. Reasons for patients’ decision to use CAM include the ability to obtain emotional support, holistic care and information from their chosen provider, as well as their perception that CAM permits patients to establish a good therapeutic relationship and cope more effectively with their medical condition(s) [7]. Other reasons include patients’ beliefs that CAM provides more personal control and a greater promise of hope than conventional therapies [6]; previous research has also found that patients appreciate what they perceive as the ease-of-access of alternatives, respect for the psycho-emotional aspects of their treatment and increased consultation time associated with CAM therapies [8].

Comparing our results of the third sequence of the meta-ethnography, the interpretation of concepts of “individualised medicine”with the ideas of research and academic medicine, politics as well as economics, we found that they differ from the current concept of the genetically and biologically oriented form of “personalised or individualised medicine”. Presently, there exists no commonly accepted definition of this form of “individualised medicine”; the lowest common denominator is actually the “division of patients (groups) by biomarkers” [4]. This contrasts considerably with the richness of humanistic issues associated with the concepts of “individualised medicine” concepts that we identified in patients reasons for seeking CAM. One dissenting aspect is the concept of “personal growth”, an effectiveness dimension which describes patients hope to be empowered by the healthcare encounter in individualised medicine. In contrast to the concepts of biomarkers and individual disease risks, the concept of the inner growth as induced by a reassessment of disease and life history can include growth in spirituality, body awareness and appreciation of nature and surroundings. In this dimension, patients request an individualised form of medicine that takes into consideration their wish for “personal growth”, including emotional disease handling. Successfully adapting to an illness or to reassess their biography in this way can enable patients to participate in social activities and feel healthy despite their physical limitations [57]. Meditation or mindfully presence in a given situation, and, consequently, the provision of such practices, can help in the search for meaning in life [58].

As an example a person-centred approach in fibromyalgia syndrome (FMS) patients of “respectfully recognizing the patients’ personal and human needs,” “encouraging the patients’ self-revelation,” “let[ting] the patient tell their story” and “digesting emotions to [patients’] illness and life situations” helped patients to identify how suffering might fit into their individual psychosocial contexts. In particular, there was a need to help patients understand how suffering might fit into family dynamics and how associated psychosocial conditions might be ameliorated [49]. Medical and therapeutic practitioners could thus be asked to support patients in their endeavour to lead a meaningful life in spite of their disease and might be urged to bear in mind that patients need therapeutic and social support to discover their resources in the personal, biographical or spiritual environment to undertake a development of inner or “personal growth” [59].

The person-centred approach in FMS noted above coincides with to the dimensions of “emotional disease handling,” “biographical reassessment” and “transformation” of our meta-synthesis in the “personal growth” concept. Moreover, in the biomedical model, diverse symptoms of diseases such as FMS are often addressed separately from their interconnectedness and linkages to the patient’s individualised bio-psychosocial factors [49]. Likewise, our concept of “personal growth” is strongly interrelated with that of “holism”, which the patients in our meta-synthesis associated with “individualised medicine”. For the patients it is important not to regard health problems in isolation; rather, they should be considered in conjunction [60]. A holistic or integrative view requires that psychological and physical treatment interdependences must work together in order to be successful [60]. In opposition to the concept of “holism”, the treatment based on individual biomarker-based stratification and genome-based information does not reflect the patients’ need to connect the disease with bio-psychosocial factors.

Also of note is that from our meta-ethnographic study it is apparent that patients like to assume responsibility for their care and that they have a wish for “personal autonomy”, which may come about via “educational empowerment” and/or “active control”. This is also manifested in patients’ desire for knowledge-building in matters of their disease. The wish of patients for “self-activation” is also related to triggering intrinsic self-healing capacities by supporting the immune system and mental health resources, as expressed in the subtheme of “activation of self-healing power”.

In contrast, the genome-based individualised healthcare that is becoming more prominent in today’s traditional medical fields connects patients’ own activity more with extrinsic factors by avoiding genetic or metabolic risks. In the patients’ view of individualized medicine with regard to “self–activation”, CAM was perceived by patients as allowing for “individual responsibility for health” [61]. Also, according to Kienle et al. (2011), patients seek CAM therapies with the aim to support and stimulate auto-protective and (auto-)salutogenic potentials, mostly with the active cooperation of the patient or of his/her body [62]. Healthcare providers must consider patients’ own experience and own body knowledge as important information. The salutogenic potential as “enabling the patient to swim” stands for the mobilisation of individual resources for more autonomy [42,62], which can be comparably expressed as the dimension of “personal autonomy” in our meta-ethnography results. The determination of individual disease risks as one goal of genome-based individualised medicine with its preliminary fixing to a possible disease does not consider the mobilisation of individual biological, psychosocial and spiritual resources.

Interestingly, as reflected in our study, a portion of what is normally called the placebo effect may be attributed to the “activation of self-healing power,”—a fact often neglected and not considered in the concept of disease risk determination. Another dimension of “personal autonomy,” namely, “educational empowerment” is a reason for the appeal of complementary medicine [63]. Lay people suffer from the circumstance that detailed technological advances in medicine have prohibited them from acquiring knowledge about their medical diagnosis [63]. Researchers potentially investigate and collect results of individuals’ biomarker-based stratification and genome-based health-related characteristics only. The knowledge and actions required for maintaining health may be controlled by persons other than individual patients who, in contrast, want to be empowered for their own health [64], as expressed in patients’ stated desire for “activation of self-healing power”.

“Self-activation” coincides here with the third-order concept of “alliance”, which reflects the subthemes of “time” and “healing relationship” in the context of the doctor-patient-interaction. These subthemes are often referred to by patients as core features for individualised care and as motivation to visit CAM providers. Thus, it should be ensured that “speaking medicine” (i.e., doctor-patient interaction), which includes the time a physician needs for detailed information and guidance is sufficiently covered by insurers and other medical health-payment systems.

Other studies show also that a patient-centred communication style of COM physicians is rated as “very important” by patients [65] and the provision of sufficient information and shared decision-making options are top patient priorities [66]. Another example, this one of personalised health care for patients with spinal cord injury, demonstrated that when a closer relationship with staff was formed, the healthcare professionals became an essential support factor; this study also found that providing patients with explicit information of patients about their condition and prognosis was necessary for their accepting the realities of their injury [48].

Consultations that last longer are perceived as being associated with a patient-centred communication style, or as a “doctor’s interest in you as a person” [48,65,66], enabling patients to realise “educational empowerment” as expressed through the concept of “self–activation”. In the view of genome-based individualised medicine, it could be debated whether the idea of a commercially available determination of risk factors through genetic diagnostic measurements empowers the individuals to seek more knowledge about their own genomes, in turn enabling them to encourage their doctors to also consider this information. The effective use of such diagnostic tools could empower patients to work with their healthcare providers to determine the most suitable prevention or treatment plan [67].

Furthermore, the findings from our meta-ethnographic study show that patients perceive medicine as highly individualised and personalised when they are able to connect different treatment options according to their own personal preferences; this is expressed in our third-order concept of “integrative care”. Here, this concept is associated with the “alliance” concept and the subtheme of establishing a “healing relationship”. “Healing relationship” stands also for shared decision making in treatment agendas integrating COM and CAM. The process of shared decision making is currently the most discussed way to take into account individual preferences. However it must be noted, that complementary treatment options are still neglected in the development of decision aids [68], although patients prefer to integrate CAM into their “tailored care” to manage their individual medical conditions [69]. Again, in this context the link between “individualised medicine” and “integrative care” can be detected [1]. One of the greatest skills of a doctor is individualisation, including subtle changes to therapy and how this therapy is delivered by a skilled healthcare provider. This influences the subjective patient’s response. A therapist who tailors his treatment will have better patients’ outcomes because she or he can more effectively embrace the meaning of the therapeutic response [70]. Over and above that, “integrative care”, including both CAM and conventional therapies for chronic diseases, could have the potential to improve a costly and fragmented delivery system [47].

On the other hand “tailored care” can coincide with gene-based risk information or tests that are customised to personal biological characteristics. Genome-based diagnostic measurements - and, consequently accurate diagnosis, specific treatments and adjusted medication doses - correlate closely with patients’ perspective of “tailored care”. However, there is a need for comprehensible information on the results of such measurements and the meaning of the diagnosis; patients need physicians to provide a medical explanation for lay people. With educational support, patients even prefer to calculate and interpret event rates and the number needed to treat or to harm [71]. We argue that gene-based risk information must therefore be accompanied by the concept of “educational empowerment”. A central dimension of “educational empowerment” is the provision of evidence based patient information which enables patients to judge and to decide according to their own preferences [71,72].

The final third-order concept of individualised medicine “wellbeing” as discussed in our study is often mentioned in the included literature as the desire for both psychological and physical “wellbeing”. Patients expressed a strong desire for individualised care provided in a familiar environment. When such care was not available, patients found it difficult to meet even basic physical needs [73]. A more familiar and less clinically medicalised environment is thus reflected as individualised care [48]. Patients seek CAM therapies as comparatively harmless ways to support the body’s healing capabilities [70,74]. The patients in our synthesised studies also sought support for the sometimes difficult work of emotional self-regulation in the dimension of “wellbeing after emotional clearing”.

The provision of functional ability is regarded as a fundamental part of “physical wellbeing”. Here, the bio-molecular concepts of differential interventions offers effective treatment and the reduction of side effects as well as unique therapeutic items (e.g., prostheses, implants adapted as a truly individual), those enable patients to continue engaging in normal activities in a sense of “wellbeing”. Moreover, regarding the desire for fewer side effects, patients’ expectations merge with the goals of genome-based individualised medicine in the search for an exact diagnosis and targeted treatment. It could be debated that the introduction of pharmacogenomic concepts into the practice of herbal medicine could be effective in reducing incidences of CAM-associated therapy failures. Furthermore, the phenomenon of psychosocial genomics, which explores the sophisticated relationship between gene expression, neurogenesis and healing practices, has the potential to reconcile biomedicine with various healing experiences brought about CAM [75].

In summary, the patients described in the included qualitative studies have a *humanistic* concept of “individualised” medicine that entails much more than individualised specifications on the molecular level, such as is the case in genome-based “personalised medicine”. Similar to the above-discussed patients’ concepts of “individualised medicine”, the German Bundestag’s report on the future of individualised medicine reflects our finding that the patients may have other preferences (e.g., emotional dimension, handling of the disease) than the genome-based concepts [3]. In addition, a clear distinction has been defined, namely that “individual medicine does not have any contribution for disease handling and the particular psychological burden which the probabilistic-predictive information of the individual medicine implies” [3]. With this statement, the report’s authors referred to the need that “individualised medicine” should be embedded in the context of “speaking medicine” (i.e., doctors-patient interaction) and psycho-social support [3].

Furthermore, in May 2012, a number of German experts discussed at the annual meeting of the German Ethics Council the expansion/addition of biologically targeted “individualised medicine” to psychological, social, biographical and spiritual aspects. In a joint effort of such medical research and care, the patient would benefit from - rather than being a victim - of progress [76].

### Study limitations

All of the studies included in our meta-ethnographic study investigated patients who used CAM as a complement to COM. We also included studies with focus groups interviewing non-CAM users being asked about their perception of CAM. The patients of the identified studies were mostly COM users in the beginning of their disease who turned to CAM for the reasons discussed above. Therefore, the investigated patient samples seem to be well balanced and can be interpreted as representing the “usual” patient population, as far as this is possible in such a qualitative approach. However, it must be emphasized that patients who turn to CAM modalities are more likely to seek out a healthy lifestyle or preventive measures than non-CAM users [77].

We must also consider that some of the concepts discussed in this study may overestimate patients’ individual perspectives as compared to the whole patient population. However, as the general trend towards more complementary and integrative health care is increasingly acknowledged as an expression of what is felt to be missing in COM, healthcare providers and decision makers should take these needs seriously as they seek to develop a modern concept of individualised medicine compatible with patients’ needs.

## Conclusions

Based on the results of our meta-ethnographic study, it can be stated that there exists a difference between the concept of individualisation from the patient perspective and the present notion of “personalised or individualised medicine” on the basis of genetics and biology. Patients’ core expectations for individualised care are a respect for “personal growth”, a “holistic” focus, a doctor-patient-“alliance”, “self-activation”, “integrative care” and “wellbeing”. There is a congruence of patients’ expectations with the goals of genome-based individualised medicine in the search for a reduction of side effects and functional ability, which would in turn enable patients to continue engaging in normal activities. In addition, detailed diagnostic measurements and consequently suited treatments, as well as adjusted medication doses correlated closely with patients’ perspective of “tailored care”. Furthermore, patients’ knowledge of genomic risk factors could be reflected their concept of “educational empowerment”.

At present, alternative other patient ideas related to individualised medicine are rarely reflected in genome-based individualisation concepts. At the individual level of patient perceptions, the concepts of individualised and integrated medicine merge. For these reasons, a comprehensive concept of “individualised and integrative health care” could be formed to include both the genome-based perspective of individualised medicine and the more holistic perspective of individualisation frequently expressed by patients. Such a comprehensive approach to medicine would provide patients the opportunity to share their commitment to “personal growth” with their healthcare provider, as well as a “holistic” view and a willingness to engage in “self-activation” with “educational empowerment”; this approach could be characterized by a doctor-patient “alliance” in the sense of “time” and the “healing relationship” and the freedom of “integrative care” and “wellbeing” through fewer side effects and increased functional ability. When allocating funds for research and health budgets, patients’ notions with regard to individual treatment should play an important role in the pursuit of a high-quality healthcare system.

## Competing interests

The authors declare that they have no competing interests.

## Authors’ contributions

All authors contributed to the development of the manuscript. All authors read and approved the final manuscript.

## Pre-publication history

The pre-publication history for this paper can be accessed here:

http://www.biomedcentral.com/1472-6882/13/124/prepub

## Supplementary Material

Additional file 1 PRISMA 2009 Checklist. Click here for file

Additional file 2 PRISMA 2009 Flow Diagram. Click here for file
