# Supplementary material for: Individualised medicine from the perspectives of patients using complementary therapies: a meta-ethnography approach
Source: BMC Complement Altern Med. 2013 Jun 3;13:124. doi: 10.1186/1472-6882-13-124 (PMC3679787; doi:10.1186/1472-6882-13-124)
Supplement: Additional file 2 — PRISMA 2009 Flow Diagram. [file 1472-6882-13-124-S2.doc]

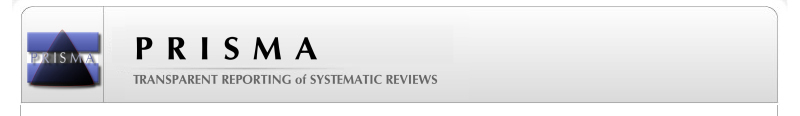
**PRISMA 2009 Flow Diagram**

**Screening**

**Included**

**Eligibility**

**Identification**

Records identified through database searching
(n = 9.578 )

Additional records identified through other sources
(n = 2 )

Records after duplicates removed
(n = 3.615 )

Records screened
(n = 223 )

Records excluded
(n = 160 )

Full-text articles assessed for eligibility
(n = 63 )

33 Full-text articles excluded, with reasons

**No reasons for CAM 14**

**Report not comprehensive enough 6**

**Secondary publication 2**

**Qualitative study with care provider 2**

**Methods presentation 1**

**Quality appraisal:**

**Results not comprehensive, plausible 4**

**No saturation 2**

**Data evaluation, analyze not clear 2**

Studies included in qualitative synthesis
(n = 30 )
